# Physiotherapy in the Polyclinic during Tokyo 2020 Olympic Games: A Detailed Analysis of Care Provided for 808 Athletes

**DOI:** 10.1298/ptr.E10332

**Published:** 2025-03-13

**Authors:** Marie-Elaine GRANT, Kathrin STEFFEN, Debbie PALMER, Torbjørn SOLIGARD, Alexandre Dias LOPES

**Affiliations:** 1 Institute of Sport and Health, University College Dublin, Ireland; 2 International Olympic Committee Medical and Scientific Commission (Games Group), Switzerland; 3 Oslo Sports Trauma Research Centre, Department of Sports Medicine, Norwegian School of Sport Sciences, Norway; 4 Edinburgh Sports Medicine Research Network, Institute for Sport, PE and Health Sciences, University of Edinburgh, UK; 5 UK Collaborating Centre on Injury and Illness Prevention in Sport, UK; 6 Medical and Scientific Department, International Olympic Committee, Switzerland; 7 Department of Physical Therapy, Northeastern University, United States of America

**Keywords:** Athlete, Rehabilitation, Sport

## Abstract

Objectives: The study provided a comprehensive analysis exploring the association between athletes’ injury characteristics and the choice of physiotherapy treatments applied at the Polyclinic Tokyo 2020. Methods: Data from all physiotherapy treatments offered in the Polyclinic Olympic Village in Tokyo between 13 July and 10 August were recorded electronically. The physiotherapy team consisted of approximately 150 Japanese physiotherapists. Physiotherapy modalities analyzed were cryotherapy, electrotherapy, exercise therapy, laser therapy, manual therapy, shockwave therapy, taping, and ultrasound therapy. Logistic regression assessed the association between athletes’ injury characteristics and the physiotherapy treatment the Polyclinic Physiotherapy Services offered at the Tokyo 2020 Olympic Games using odds ratio. Results: This study analyzed 808 athletes who received physiotherapy, of which 66.6% (n = 1023) were for lower limb chronic injuries affecting muscles and tendons (72.8%, n = 1209) and were the most prevalent ones treated. Chronic injuries accounted for 56.7% (n = 942). Manual therapy was associated with trunk injuries (odds ratio 1.8, 95% confidence interval 1.4–2.3) and chronic injuries (1.5, 1.2–1.9). Ultrasound therapy was associated with injuries of the upper limbs (3.6, 1.9–6.7) and lower limbs (3.0, 1.7–5.2). Taping was associated with bone/joint/ligament injuries (2.1, 1.4–3.0). Shockwave was associated with muscle/tendon injuries (2.0, 1.2–3.4). Cryotherapy was related to acute injuries (1.8, 1.1–3.0) and lower limbs (3.6, 1.4–9.4). Laser was associated with bone/joint/ligament injuries (14.4, 4.5–45.8). Conclusions: Chronic injuries affecting lower limb muscles and tendons were the most prevalent musculoskeletal injuries treated at the Tokyo 2020 Polyclinic. Overall, Japanese physiotherapists used manual therapy, taping, exercise, and ultrasound therapy to treat athlete injuries.

## Introduction

Physiotherapy was important during the 2020 Tokyo Games in athlete support and providing excellence in care. The IOC (International Olympic Committee) recognizes that the role of the Physiotherapist is essential to the success of a multidisciplinary sports medicine team^1)^. Many National Olympic Committees (NOCs) include physiotherapists on their accredited medical teams to the Olympic Village and competition venues. The Tokyo Organising Committee of the Olympic Games (TOCOG) and Paralympic Games also made physiotherapy services available at the Polyclinic in the Olympic Athletes’ Village, providing all athletes easy access to physiotherapy and healthcare services^1)^. This is especially important for athletes who do not have their own NOC physiotherapy support and also offers athletes additional treatment options. The Polyclinic offers convenient and prompt investigation and treatment in a state-of-the-art outpatient medical facility for all athletes.

The field of sports medicine has traditionally emphasized the importance of relying on the best available scientific evidence to inform treatment decisions for athletes who have sustained injuries^2)^. However, socio-cultural factors can also significantly shape health professionals’ approach to managing injuries^3)^ in a multidisciplinary environment. There is a gap in the literature, with a limited number of studies exploring the choice of physiotherapy treatment approach within the sports medicine community, particularly among physiotherapists. Previous studies outline the utilization of physiotherapy services at the polyclinic during the Olympic Games^1,4,5)^. The important information offered by these previous studies focused on descriptive analysis. Thus, the present study sought to provide a comprehensive analysis exploring the association between athletes’ injury characteristics and the choice of physiotherapy treatments applied at the Polyclinic Tokyo 2020.

## Methods

The TOCOG provided physiotherapy and related services embedded in the Polyclinic located in the Olympic Village in Tokyo and at sports venues. TOCOG’s services were available for all 11315 participating athletes from 206 NOCs and also extended to coaches, trainers, team managers, the TOCOG workforce, and the Olympic family. The Polyclinic was open for a total of 30 days from the “pre-competition period” (13 July to 22 July, 10 days), the “duration of Olympic competitions” (23 July to 8 August, 16 days), and 2 days of “post-competition until the 10 August.” All physiotherapy treatments offered in the Polyclinic Olympic Village in Tokyo between 13 July and 10 August were analyzed in this study (treatments provided at sports venues or by national teams were not included). The collection and use of data in this study were in accordance with the IOC Olympic Movement Medical Code, which requires athletes to provide voluntary and informed consent for any medical intervention, including physiotherapies, and for their data to be anonymized for research purposes. All information was treated confidentially, and the database was de-identified following the Games. The study received approval from the Medical Research Ethics Committee of the South-Eastern Norway Regional Health Authority (2011/388) and the OCOG medical advisory group.

### Physiotherapy team

To provide Olympic Physiotherapy services for the Tokyo 2020 Olympic Games, 750 Physiotherapists were required, 150 for the Polyclinics and 600 for the Competition Venues.

All physiotherapists were required to be members of the Japanese Physical Therapy Association (JPTA) with a BSc in Physiotherapy. Minimally 3 years post-graduate experience was required, and 5 years to be a member of the Field of Play team or to provide cover at sports competition venues. The JPTA received approximately 1000 applications, forwarding 650 candidates to TOCOG after initial screening. About 600 were selected for online interviews, of which 500 were selected, with an additional 110 recommended by national federations. A sports physiotherapy training program was developed by the JPTA’s education advisory board, which also provided funding. The program included 40 hours of training in sports taping, manual techniques, emergency care, cryotherapy, electrotherapy, and injury assessment. Despite COVID-19 challenges, the program successfully trained 450 physiotherapists, who were then included in the staff recruitment for TOCOG in collaboration with the JPTA. This training initiative was highly successful and is considered one of the legacies of the Tokyo Olympic Games. Each member of the physiotherapy team volunteered for 10 days.

At previous games, international volunteer physiotherapists have been recruited onto the host nations’ Physiotherapy team at the Policlinic which is encouraged by the IOC to meet an international approach to care of athletes from all around the globe. Because of the COVID-19 pandemic and the associated restrictions, it was not possible for the TOCOG to recruit international physiotherapists onto the team. Therefore, the Tokyo 2020 physiotherapy team was composed exclusively of Japanese physiotherapists, with no international physiotherapists. All physiotherapists were required to have at least 3 years of postgraduate experience, membership in the JPTA, and a license to practice in Japan. Three years prior to the Tokyo 2020 Olympic Games, JPTA had recognized a need for further sports physiotherapy training for physiotherapists to meet athletes’ requirements during the Olympic Games. In addition to physiotherapists, sports massage therapists with a combined qualification in acupuncture were included as part of the physiotherapy team. However, this research exclusively examined therapies administered by physiotherapists.

### Referral procedures

All injuries and first-time reasons to attend physiotherapy were required by Japanese law to be first assessed by and receive a referral from a medical doctor licensed to practice in Japan. Follow-up treatments did not require new referrals on every attendance once an initial referral had been provided. However, physiotherapists could conduct assessments, make diagnoses, and prescribe interventions without any constraints.

### Physiotherapy encounter record

The electronic form included detailed information on injuries, such as affected body parts, type of injury, injury onset, and the physiotherapy treatment offered. The form was available in Japanese and English. Encounter was defined as a single polyclinic visit, which may involve one or several treatment modalities provided during the same visit.

### Physiotherapy services at Polyclinic

Athletes had a diverse range of physiotherapy modalities available to them at the polyclinic physiotherapy department for the treatment of injuries and to support performance and recovery. An extensive range of equipment (e.g., electro-biofeedback, electromagnetic field, ultrasound therapy, laser, shockwave) was available. In addition, a wide range of taping, rehabilitation, recovery, exercise, and cold therapy equipment, including ice-baths, was available. This study categorized 8 physiotherapy treatment interventions: (1) cryotherapy, the application of ice used in the treatment and management of injury for pain relief and reduction of inflammation; (2) electrotherapy, which includes the use of interferential current, electrical muscle stimulation, high-voltage-pulsed galvanic stimulation, transcutaneous electrical nerve stimulation (TENS), microcurrent, and ultrashort wave (radio wave). These modalities are usually used to promote muscle function, reduce pain, and promote healing; (3) exercise therapy, the use of physical exercises and activities to restore, maintain, or improve physical function; (4) laser therapy, low-level laser therapy or cold laser therapy, involves the application of low-level lasers or light-emitting diodes usually used to stimulate tissue repair, and alleviate pain; (5) manual therapy, including joint mobilization and manipulation, soft tissue manipulation, myofascial release, trigger point, and massage techniques; (6) shockwave or extracorporeal shockwave therapy involves the application of controlled extracorporeal shockwave to the affected area, aiming to, reduce pain, and promote tissue repair; (7) taping, application of specialized adhesive tapes to support, stabilize, or protect specific joints, muscles, or ligaments; and (8) ultrasound therapy, application of high-frequency sound waves to promote repair and reduce pain in soft tissue injuries. A full description of the area, facilities, and equipment is presented in Supplemental File 1.

### Analysis

Descriptive statistics were used to describe characteristics of the injuries treated and physiotherapy treatment used by the Physiotherapy Service at Polyclinic Tokyo 2020. We performed a logistic regression to assess the association between athletes’ injury characteristics and the physiotherapy treatment. The results were presented as odds ratio (OR) and their respective 95% confidence intervals (95% CI). Results were significant when 95% CI did not cross 1.0. All analyses were performed using SPSS 27.0 for Windows and are consistent with the CHecklist for Statistical Assessment of Medical Papers (CHAMP) statement^6)^.

## Results

**Fig. 1.**
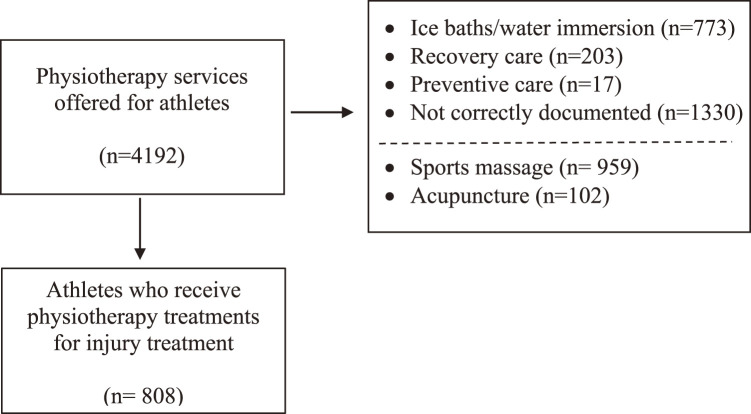
Physiotherapy services by number of athletes at the Polyclinic Tokyo 2020

A total of 4192 athletes received physiotherapy treatment in the Polyclinic during the Games. Out of the 3384 encounters analyzed, 2054 were not related to injury treatment and 1330 were not fully documented. Hence, this study focused on the analysis of the treatment of injuries for 808 athletes. As athletes could receive multiple physiotherapy treatments during the same session or encounter, each athlete received an average of 2.02 treatments per encounter (range: 1–3 physiotherapy treatments). This study examined 1651 physiotherapy treatments provided to 808 athletes (Fig. 1).

### Characteristics of injuries treated by physiotherapists at the Polyclinic

Athletes seeking physiotherapy exhibited a varied pattern of injuries. The lower limb was the primary anatomic location of injuries (66.6%, n = 1023). Muscle and tendons were the main locations of injuries observed (72.8%, n = 1209). Chronic injuries accounted for 56.7% (n = 942) of the injuries treated. Table 1 presents a detailed description of the location and types of injuries treated by physiotherapists at the Polyclinic.

**Table 1.** Characteristics of the injuries treated by the Physiotherapy Service at Polyclinic

Injury characteristic	Sessions of treatment
Injury anatomic location	
Trunk/Abdomen	23.5% (388)
Upper limb	14.5% (240)
Lower limb	61.9% (1023)
	100% (1651)
Type of injury	
Bone/Joint/Ligament	25.1% (406)
Muscle/Tendon	74.8% (1209)
	100% (1615)
Injury onset	
Acute	40.1% (666)
Chronic	56.7% (942)
	100% (1608)

Categorical data variables are expressed in percentage and number of treatments. Please note the different totals for acute and chronic injuries because some records did not report this information.

### Physiotherapy treatments used to treat injuries at the Polyclinic Tokyo 2020

Manual therapy emerged as the predominant intervention, consistently applied regardless of the anatomic location, anatomical structure, or type of injury, constituting a substantial portion of the range. A comprehensive breakdown of the physiotherapy treatments offered based on injury characteristics at Polyclinic Tokyo 2020 is detailed in Table 2. Significant statistical associations were identified in relation to anatomic location and manual therapy, ultrasound therapy, and cryotherapy. When examining the association between the injured anatomical structure and the treatment administered, taping, shockwave, and laser therapies displayed statistically significant associations. Statistically significant associations were also found between the type of injury (acute or chronic) and manual therapy, ultrasound therapy, cryotherapy, and laser therapy. A detailed analysis of the correlation between athletes’ injuries and the corresponding physiotherapy treatments at Polyclinic Tokyo 2020 is presented in Table 3.

**Table 2.** Physiotherapy treatment by characteristics of the injuries treated by the Physiotherapy Service at Polyclinic Tokyo 2020

	Manual therapy	Taping	Ultrasound therapy	Exercise therapy	Electrotherapy	Shockwave	Cryotherapy	Laser
Anatomic location								
Trunk/Abdomen	45.5 (176)	5.9 (27)	16 (62)	16.3 (74)	10.3 (40)	1.8 (8)	1.3 (5)	0.4 (2)
Upper limb	32.5 (77)	16.7 (40)	13 (31)	15.1 (36)	4.6 (11)	13.0 (31)	4.2 (10)	1.3 (3)
Lower limb	32.5 (332)	16.4 (168)	12.5 (128)	12.1 (124)	10.4 (106)	10 (102)	4.7 (48)	1.5 (15)
Anatomical structure								
Bone/Joint/ Ligament	32.3 (131)	15.3 (62)	10.4 (42)	13.3 (54)	4.9 (20)	15.6 (63)	4.2 (17)	4.0 (16)
Muscle/Tendon	36.5 (441)	16.6 (201)	14.2 (171)	10.2 (123)	11.1 (134)	7.2 (87)	3.8 (46)	0.4 (5)
Type of injury								
Acute	30.6 (203)	17.0 (113)	12.2 (81)	12.2 (81)	10.4 (69)	12.3 (82)	5.1 (34)	0.2 (1)
Chronic	39.2 (369)	15.6 (147)	14.0 (132)	10.3 (97)	9.0 (85)	6.8 (64)	3.0 (28)	2.1 (20)

Data variables are expressed in percentage and number of participants.

**Table 3.** Association between athletes’ injury characteristics and the physiotherapy treatment offered

	Manual therapy	Taping	Ultrasound therapy	Exercise therapy	Electrotherapy	Shockwave	Cryotherapy	Laser
Anatomic location								
Trunk/Abdomen	1.838* (1.427–2.368)	1	1	1	1	1	1	1
Upper limb	1.038 (0.760–1.419)	2.015* (1.052–3.862)	3.602* (1.913–6.781)	0.904 (0.554–1.477)	1.161 (0.730–1.847)	0.539 (0.265–1.096)	3.006 (0.985–9.173)	0.395 (0.066–2.351)
Lower limb	1	2.023* (1.179–3.470)	3.057* (1.782–5.245)	0.744 (0.526–1.052)	1.096 (0.781–1.537)	1.142 (0.764–1.705)	3.696* (1.451–9.417)	0.534 (0.128–2.226)
Anatomic structure								
Bone/Joint/ Ligament	1	2.104* (1.450–3.051)	1	1	1	1	1	14.495* (4.586–45.819)
Muscle/Tendon	1.088 (0.840–1.408)	1	0.886 (0.619–1.270)	1.455 (0.986–2.145)	1.127 (0.810–1.569)	2.090* (1.271–3.435)	1.003 (0.555–1.813)	1
Type of injury								
Acute	1	1.902* (1.339–2.703)	1	1	1	1	1.852* (1.108–3.096)	1
Chronic	1.551* (1.248–1.928)	1	0.821 (0.596–1.132)	1.182 (0.872–1.601)	0.845 (0.644–1.110)	0.840 (0.597–1.181)	1	17.354* (2.263–133.110)

The reference was modified to facilitate data interpretation. The results are presented as odds ratio and their respective 95% confidence interval.

*Statistical significant association. “1” are reference values, for which there is one for each treatment modality.

## Discussion

The purpose of this investigation was to provide a comprehensive analysis exploring the association between athletes’ injury characteristics and the physiotherapy treatments applied at the Polyclinic Tokyo 2020. Overall, the study analyzed 808 athletes who underwent 1633 physiotherapy treatments at Polyclinic Tokyo 2020 for their injuries, equating each athlete to receive an average of 2 treatments. This is similar to findings at the Rio 2016 Olympic Games^5)^ with an average of 2.5 treatments per athlete encounter. Athletes seeking physiotherapy presented with a range of injuries, with the lower limb as the predominant anatomic location, comprising two-thirds of all injuries, and muscles/tendons as the primary affected structures, constituting nearly one-fourth of all injuries^5)^. The study on physiotherapy services provided during the 2004 Olympic Games^4)^ in Athens revealed comparable findings, demonstrating that 75% of treated injuries were associated with the lower limb, with muscles/tendons being the primary affected anatomical site. By contrast, the examination of physiotherapy services during the 2012 London Games^1)^ indicated that roughly 50% of injuries involved muscles/tendons, and the trunk/column accounted for 44.9%, differing from our study’s anatomical location findings of 75% associated with the muscles/tendons. Despite variations in injury classification and anatomical location criteria between Tokyo and previous Games in Athens and London^1,4)^, a consistent injury type and location pattern emerged across these three Olympic Games.

Manual therapy was the most common treatment modality consistently administered irrespective of anatomic location, anatomical structure, or injury type. In Athens 2004^4)^, manual therapy was one of the most common treatment modalities used (approximately 15%), and in London 2012^1)^, it represented the most common treatment offered by physiotherapists (21.8%). In Rio 2016^5)^, a similar pattern was observed where mobilization techniques, manipulative, and soft tissue techniques accounted for 53% of the treatments administered. Manual therapy, taping, ultrasound therapy, and exercise therapy constituted the primary treatments administered by the physiotherapists at Polyclinic Tokyo 2020. This finding aligns with the treatment patterns observed in Athens 2004^4)^ and London 2012^1)^. In Athens 2004^4)^, the main treatments included ultrasound therapy, manual therapy, and exercise therapy, while London 2012^1)^ featured manual therapy, taping, and exercise therapy as the primary interventions. Notably, ultrasound therapy, previously prominent in Athens, did not rank among the top 5 modalities in London 2012^1)^. In Rio 2016^5)^, the primary interventions (excluding ice cold baths) were manual therapy and electrotherapy. However, in Tokyo 2020, ultrasound therapy was seen as the primary electrotherapy intervention employed by physiotherapists. Modality shifts across the Olympic Games may result from either the prevailing physiotherapy practices in line with international standards or the adoption of the latest evidence-based approaches.

The odds of receiving manual therapy for injuries in the trunk/abdomen were 1.8 times higher compared with the lower limb. There is a notably higher likelihood (1.5 times) of using manual therapy for chronic over acute conditions. Manual therapy included therapeutic massage, mobilization, and manipulation. The utilization of manual therapy in sports physiotherapy is a topic of debate, frequently drawing criticism due to the absence of conclusive evidence endorsing its efficacy^7)^. Moderate- to low-quality evidence suggests that manual therapy can improve symptoms in athletes when used alone^8)^. However, it is important to highlight that the beneficial effects of this therapy are limited to immediate- and short-term durations, aligning with the requirements of athletes seeking treatment for injuries and conditions during high-performance events such as the Olympics.

The odds of receiving taping for injuries in the upper and lower limbs were approximately 2 times higher compared with a trunk. For injuries of the bone, joint, or ligament, the odds of receiving taping (treatment) were 2.1 times higher than for injuries of muscle/tendon. Acute injuries showed a 1.9 times higher probability of being taped compared to chronic injuries. Taping remains the method of choice for athletes, particularly for joint or ligament injuries in the lower limb, as it is used extensively for athletes with lateral ankle injuries^9)^. The extensive use of taping techniques can be attributed to each area’s unique anatomical and biomechanical considerations. Lower limb injuries often require additional targeted support and stability to facilitate healing, prevent further damage, reduce excessive joint movement, and alleviate stress on injured structures to enhance mobility and function^9)^.

Some interesting results were observed when verifying the association between athletes’ injury characteristics and the physiotherapy treatment. Acute injuries were found to have a 1.8 times higher probability of receiving cryotherapy in comparison to chronic injuries. This preference can be explained because cryotherapy is used to treat and manage injury, which is thought to help reduce pain and inflammation^10)^. Injuries located in the lower limb are 3.6 times more likely to undergo cryotherapy compared to those in the trunk/abdomen. This could be attributed to the practical challenges of applying ice packs to the trunk/abdomen region. Injuries in muscle/tendon are twice as likely to undergo treatment with shockwave therapy compared to injuries affecting bone/joint/ligaments. This can be attributed to the widespread use of shockwave therapy in treating tendinopathy^11)^. Despite numerous studies, including systematic reviews^12,13)^, demonstrating the efficacy of laser therapy, this modality was not used extensively. Chronic injuries, primarily concentrated in the lower limb, displayed a heightened inclination for undergoing laser treatment in the few instances where laser therapy was applied, in contrast to acute injuries and muscle/tendon injuries.

While the emphasis in sports medicine has predominantly been on identifying the best available scientific evidence, there is a notable scarcity of studies addressing how the culture of physiotherapists, physicians, and other relevant professionals can shape and influence the treatment choices for injured athletes^2)^. This study brings a unique opportunity to understand how approximately 250 physiotherapists from the same country (Japan) treated 808 athletes within a shared service and space in the Tokyo 2020 Polyclinic. All therapists had at least 3 years of experience, and most underwent 40 hours of sports physiotherapy training facilitated by the JPTA. The training offered before the Tokyo 2020 could influence the results of this study, as some protocols were discussed and suggested. However, the training was conducted by physiotherapists from the same country, reflecting the approach of this Japanese physiotherapy team to sports injuries. In general, Japanese physiotherapists preferred manual therapy, taping, exercise, and ultrasound therapy to treat athletes. This inclination aligns with the range of practices seen in previous Olympic Physiotherapy services, London 2012^1)^ favored manual therapy while Rio 2016 favored both manual and electrotherapy^5)^.

This study offers valuable insights for planning physical therapy services at upcoming major sporting events and may also influence treatment approaches for physiotherapists across performance levels, including recreational athletes. Manual therapy emerged as the predominant intervention, consistently administered regardless of anatomic location, anatomical structure, or injury type. Ultrasound therapy was used in injuries located mainly in the limbs. Cryotherapy was predominantly used for acute lower limb conditions, shockwave therapy-targeted muscle/tendon injuries, and laser therapy was primarily employed for chronic lower limb injuries.

The main limitation of this study was the elevated number of missing data entries in the electronic form. The electronic form utilized by the physiotherapists was adapted from an Electronic Medical Record, which may have influenced the quality of the recorded physiotherapy treatment information. Lastly, the COVID restrictions implemented during the event could also affect the treatment approach.

## Conclusion

Chronic injuries affecting the lower limb muscles and/or tendons were the most prevalent issues treated by physiotherapists at the Polyclinic Tokyo 2020. Overall, Japanese physiotherapists preferred employing manual therapy, taping, exercise, and ultrasound therapy to treat athletes.

## Acknowledgments

We extend our deepest gratitude to the outstanding physiotherapists whose expertise and dedication were instrumental in the success of this research. Without the meticulous oversight of physiotherapy services and data collection by Masaki Katayose (Lead Physiotherapist TOCOG), Tatsuya Tamaki, Miwako Tohyama (senior members TOCOG Physiotherapy team), this study would not have been possible. Their invaluable contributions and commitment to excellence are sincerely appreciated.

## Funding

This research did not receive funding from any specific public, commercial, or non-profit organization.

## Conflicts of Interest

There is no conflicts of interest to disclose.

## Supplementary Material

Tokyo 2020 Olympic Games Main Polyclinic in the Olympic Village at Harumi: Physiotherapy Department.
