# Supplementary material for: Physiotherapy in the Polyclinic during Tokyo 2020 Olympic Games: A Detailed Analysis of Care Provided for 808 Athletes
Source: Phys Ther Res. 2025 Mar 13;28(1):61–7. doi: 10.1298/ptr.E10332 (PMC12047046; doi:10.1298/ptr.E10332)

Tokyo 2020 Olympic Games

Main Polyclinic in the Olympic Village at Harumi: Physiotherapy Department

Description of the Area and Facilities

There were three polyclinics planned for the Tokyo 2020 Olympic Games – each incorporated a physiotherapy department:

1. The polyclinic in the main Olympic Village at Harumi.
2. Satellite polyclinic, physiotherapy department at the Sailing Olympic Village at Oiso.
3. Satellite polyclinic physiotherapy department at the Cycling Olympic Village at Izu.

For the purposes of this paper the data analysed is based on the physiotherapy activity at the main Polyclinic in Harumi.

Spatial provision at the main physiotherapy department in the Harumi Olympic village.

The physiotherapy department in the polyclinic in the Harumi Olympic village was situated at ground floor level near the entrance of the polyclinic. It was well sign posted and easily accessed by athletes.

The spatial requirement for the physiotherapy and physical therapies department was approximately 850m^2

The physiotherapy and physical therapies department was divided into 5 main areas:

1. **Reception area** (approximately 30m^2)
   The reception area provided a reception desk at the entrance to the department accommodating three PCs with access to the EMR system.

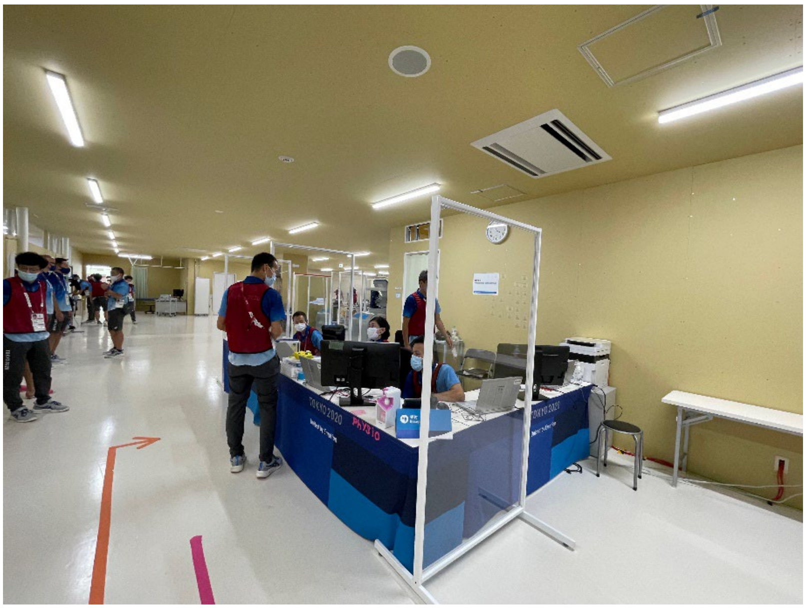

2. **Treatment Area** (approximately 300m^2)

The main physiotherapy treatment area included:

- 8 treatment cubicles screened off by synthetic curtain.
- An extensive range of electrotherapy equipment (see Equipment List Attached) which were placed on suitable mobile equipment trolleys.
- The general treatment area also incorporated a dedicated space for wet cold therapy treatments distancing wet treatments from electrotherapy treatments. The wet area allowed for application of ice packs and cold compression Games-ready cold therapy equipment.

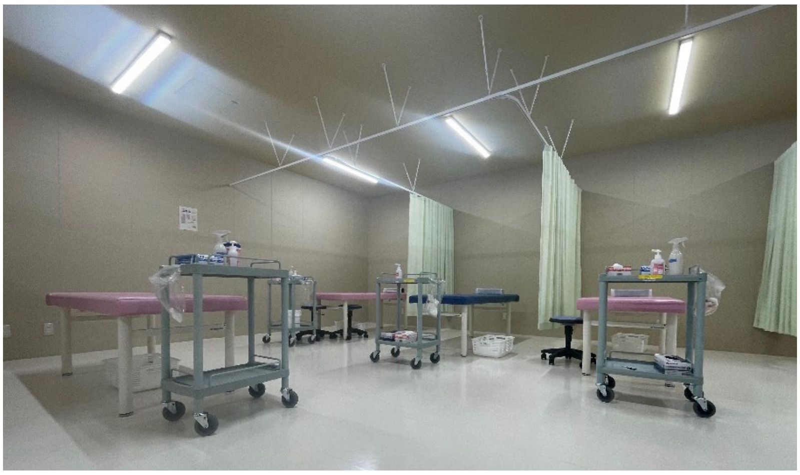

3. **Rehabilitation and Exercise Area** (approximately 50m^2)

- Space for exercise mats, Swiss balls, balance boards, foam rollers, light weights, and a wide range of elastic thera-bands for rehabilitation.
- An Alter-G anti-gravity treadmill was provided for lower limb partial weight bearing rehabilitation.

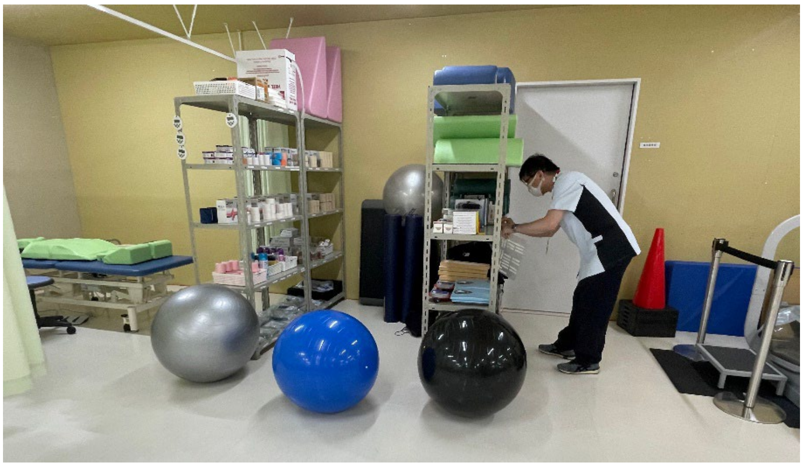

4. **The Cold Bath & Cryotherapy Area** (approximately 170m^2)

The cold bath and cryotherapy area included:

- Separate male/ female changing areas.
- Separate male/ female showers.
- 3 ice machines producing approximately 1,200kgs of cubed ice every 24 hours.
- 9 single total emersion baths each attached to a filter system, some baths sharing a filter system and others having an individual filter system.
- Each bath was attached to a chiller, circulation pump and flexible hoses with quick release connectors, continually circulating chilled water facilitated both filtering of the water to ensure maximum hygiene and maintenance of temperature to ensure maximum therapeutic effect.
- A non-slip floor with suitable drainage.
- Water supplies and hoses were available for efficient refiling of baths.
- Electronic wireless monitoring the temperature of the water of each bath.

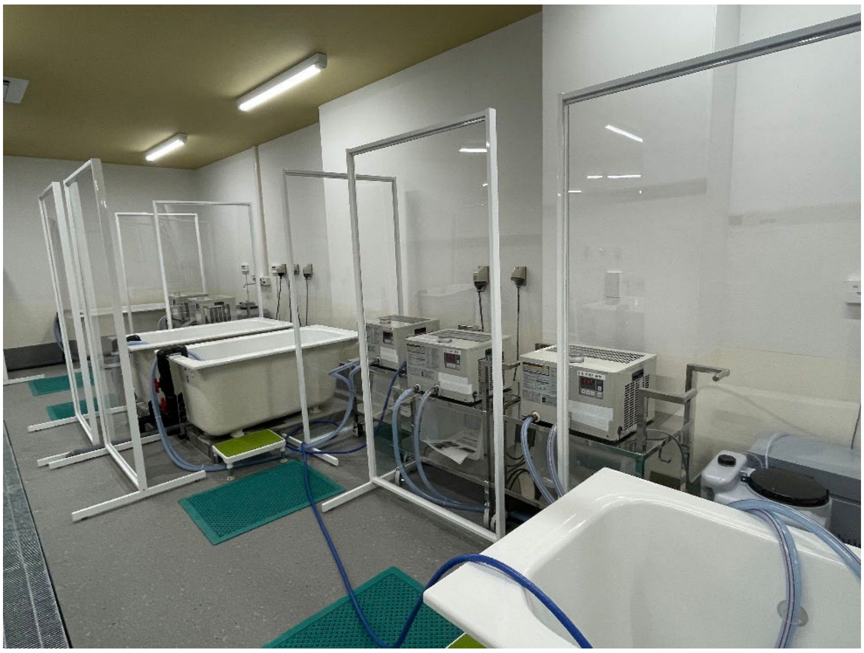

5. **Acupuncture and Massage Room** (approximately 120m^2)

The acupuncture and massage area comprised of a large treatment area which could be accessed via the physiotherapy department.

- There were three individual treatment rooms of approximately provided for in the massage and acupuncture area in the event of an athlete requiring an increased level of privacy for consultation or treatment.

- 8 acupuncture and massage cubicles with treatment tables well-spaced with appropriate screening, synthetic curtain which could be cleaned and disinfected after every treatment.
- Appropriate provision of suitable oils and massage gels.
- Appropriate provision of acupuncture needles and disposal units for acupuncture needles.

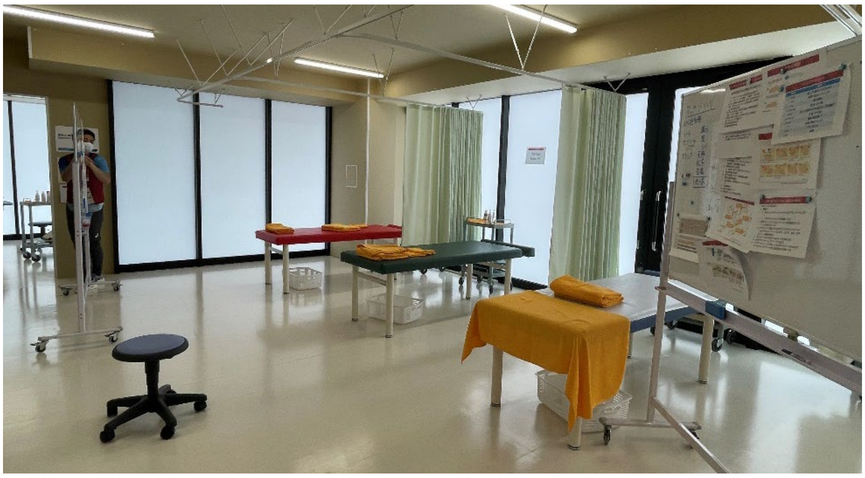

Supplement: Supplementary file 1 — Tokyo 2020 Olympic Games Main Polyclinic in the Olympic Village at Harumi: Physiotherapy Department. [file ptr-28-61-s01.pdf]
